# Elite Athletes’ Perfectionistic Striving vs. Concerns as Opposing Predictors of Self-Handicapping With the Mediating Role of Attributional Style

**DOI:** 10.3389/fpsyg.2022.862122

**Published:** 2022-05-06

**Authors:** Lilla Török, Zsolt Péter Szabó, Gábor Orosz

**Affiliations:** ^1^Unité de Recherche Pluridisciplinaire Sport Santé Société, Université d’Artois, Liévin, France; ^2^Department of Psychology and Sport Psychology, University of Physical Education, Budapest, Hungary; ^3^Department of Ergonomics and Psychology, Budapest University of Technology and Economics, Budapest, Hungary

**Keywords:** elite athlete, perfectionistic striving, perfectionistic concerns, self-handicapping, sport-related attributional style

## Abstract

Self-handicapping is not only present among amateurs, but also even among the most elite athletes. The vast majority of the research investigates self-handicapping in academic context among students with mediocre performance. However, scientific examinations of predictors among top performers in the field of sports is terra incognita. Among the predictors of self-handicapping, perfectionistic strivings, and concerns as well as attributional style, were demonstrated as relevant ones among samples in prior studies. However, these links have never been examined among elite athletes who can be characterized by various aspects of perfectionism. In this study, the link between self-handicapping and perfectionistic striving and concerns was examined both directly and indirectly through the potential mediating effect of attributional style among elite athletes (*N* = 111) where more than half of the participants was competing at international level such as European and World Championships or Olympic Games. As it was expected, a positive relationship was found between perfectionistic concerns and self-handicapping, whereas the findings suggested a negative relationship between perfectionistic striving and self-handicapping. These connections were partially mediated by attributions for negative sport-related events. It appears that explanations for negative events are crucial in connection with protecting oneself through self-handicapping even among top athletes. The present work is a first step of a broader program in which the goal is reducing self-handicapping of top athletes through attributional retraining intervention.

## Introduction

Professionals working with elite athletes often face the issue of setting excessively high standards and finding ways to prevent inherent failure. This challenge is demonstrated by the behavior of prominent and perfectionist soccer player David James (Daily Mail, 2008). In a memorable report from 1997, he revealed that instead of sleeping he had been playing video games all night the day before a critically important match against Newcastle (ESPN, 2011). Besides these anecdotes very little is known about elite athletes’ self-handicapping. In the present study, we intended to thoroughly explore a relationship pattern among multiple dimensions of perfectionism and self-handicapping, while focusing on the potential mediating effect of attributional style regarding sport-related events.

### Self-Handicapping

An athlete constantly arriving at the track minutes before competitions or complaining about injuries are both examples of self-handicapping in sport. According to Berglas and Jones (1978, p. 406) self-handicapping “involves any action or choice of performance setting that enhances the opportunity to externalize (or excuse) failure and to internalize (reasonably accept credit for) success.” In their seminar paper, the authors argued that many professional athletes might resort to self-handicapping. Self-handicappers control their attributions by providing themselves an opportunity to externalize a poor performance and protect their self-esteem. By contrast, self-handicapping has also been found to positively relate to failure avoidance (Elliot and Church, 2003).

Surprisingly, little empirical evidence is available on self-handicapping among elite athletes. Considering its negative impact on performance (Elliot et al., 2006; Schwinger et al., 2014) and specifically sport performance (Coudevylle et al., 2008), it is important to thoroughly understand the process of self-handicapping.

### Perfectionism

Perfectionism is a trait characteristic of a person striving for flawlessness and setting excessively high performance standards, accompanied by overly critical self-evaluations. It is also characterized by their concerns over how others may evaluate them (Frost et al., 1990). Adaptive (i.e., perfectionistic striving) and maladaptive (i.e., perfectionistic concerns) aspects of perfectionism are often distinguished (see Gotwals et al., 2012 for review; Frost et al., 1993; Rice and Ashby, 2007; Stoeber and Gaudreau, 2017). Perfectionistic striving is characterized by realistic and reasonable self-expectations, with attempts to strive for flawlessness accompanied by a sense of satisfaction and enhanced self-esteem. However, perfectionistic concerns involve setting unrealistically high standards and are driven by a fear of failure, while being connected to conditional approval (Terry-Short et al., 1995).

There is increasing interest in perfectionism in the realm of sport (Hill et al., 2020). As Rees et al. (2016, p. 1049) stated, “super-elite athletes are conscientious, optimistic, hopeful, and perfectionist.” While a high level of perfectionistic striving is beneficial in order to succeed (Gould et al., 2002; Gotwals et al., 2012), high levels of perfectionistic concerns relate to viewing sporting experiences negatively (Sellars et al., 2016). Gould et al. (2002) found that Olympic champions scored highly on the adaptive aspect of perfectionism but had low scores on the maladaptive form. Also, Sellars et al. (2016) reported negative impact (e.g., dissatisfaction, over-thinking, aggression, concerns over mistakes, and fear of failure) of perfectionistic concerns on sporting experiences. Despite it appears that top athletes benefit from perfectionistic striving and suffer from perfectionistic concerns, it is not well studied how these are related to their self-handicapping tendencies.

### Attributional Style

To organize, simplify and explain their experiences, people formulate causal attributions. Attributional style is a moderately stable characteristic influencing how the outcome of an event may be explained habitually by the given person (Peterson and Steen, 2002). Explanations could be classified along several attributional dimensions (e.g., locus of causality, stability, controllability, globality, and intentionality; Hanrahan et al., 1989).

It has been found that the explanatory style is associated with athletic performance. Gordon (2008) found that soccer players scoring high on optimism (with internal, stable, and global attributions to positive events) demonstrated better performance during a loss than pessimists (with external, unstable, and specific attributions to positive events). Prior studies showed that attributional style serves to preserve one’s self-esteem in case of failure (e.g., Rees et al., 2005). Despite scientific examination of attributional styles has an in-depth scientific tradition, very little is known about top sports performers’ attributions in the light of their perfectionism and self-handicapping.

### The Relationship Between Self-Handicapping, Perfectionism, and Attributional Style

Self-handicapping and the attributional theory are interconnected on several levels. According to Rhodewalt (2008), self-handicapping occurs for the purpose of controlling one’s attributions about the self. Pioneering review of Rhodewalt (1990) considered attributional style to be a key factor in understanding the dynamics of self-handicapping; however, very few and inconsistent results are available. It proposed that in comparison to low self-handicappers, high self-handicappers attribute positive daily life events to less stable and less internal factors, whereas they attribute negative events to more stable factors. The two groups did not differ in their external attributions for negative events. The study also reported no difference between the two groups in terms of the globality (and specificity) of the attributions. Another study found that self-handicapping was significantly associated with an internal, stable, and global attributional style for negative events (Rhodewalt and Hill, 1995). Regarding locus of control, Stewart and de George-Walker (2014) reported positive association between external locus of control and self-handicapping.

According to Kearns et al. (2008), perfectionism and self-handicapping go hand in hand. More specifically, Stewart and de George-Walker (2014) reported that self-handicapping and perfectionistic concerns are positively related. In the same vein, Pulford et al. (2005) reported a negative association between the adaptive aspects of perfectionism and self-handicapping. The authors argue that highly self-oriented perfectionists (predominantly adaptive in nature) in comparison to low self-oriented perfectionists care more about how they achieve their success, thus are less prone to self-handicapping.

The relationship between perfectionism and sport-related attributions has not been researched in detail. According to Anshel and Mansouri (2005), high perfectionistic concerns (i.e., concerns over mistakes) are related to internal causal attributions at the event of failure. However, they did not investigate attributions in relation to events of success, thus the methodology used remains one-sided or partial. Stoeber and Becker (2008) found that the adaptive aspects of perfectionism (i.e., striving for perfection) are positively related to self-serving attributions, whereas perfectionistic concerns (i.e., negative reactions to imperfection) were positively related to self-depreciating attributions among female soccer players. Periasamy and Ashby (2002) reported that both types of perfectionists scored higher on internal locus of control than non-perfectionists. As Stewart and de George-Walker (2014) proposed, the interaction between maladaptive social cognitive constructs and self-handicapping needs further investigation.

In the present study, we propose that perfectionism and self-handicapping are connected to each other, and that this relationship is partially mediated by the attributional style that top athletes adopt. More specifically, we propose that perfectionistic concerns positively correlate with self-handicapping, whereas perfectionistic striving negatively correlates with self-handicapping. We also expect that perfectionistic concerns positively associate with the level of internal, stable, controllable, intentional and global attributions for negative sport-related outcomes, and this partially explains their tendencies for self-handicapping.

## Materials and Methods

### Participants and Procedure

A sample of 111 elite (based on Rankinen et al., 2000; Swann et al., 2015) athletes (66 men, 45 women) participated in our study. More than half of the sample (54.9%) were medalists or participants of international competitions, such as European and World Championships or Olympic Games and 36.9% were national level competitors such as medalists (51.2% of them gold medalists) at national level (8.1% missing). Using a snowball sampling method, they were recruited to participate in an online survey *via* email from the personal contacts of the first author. The age of the participants ranged from 13 to 36 (*M* = 20.76, *SD* = 4.75). Fifty-nine participants took part in individual sports, 32 in team sports, and 20 in both individual and team sports. The time participants spent playing sports competitively was an average of 9.91 years (*SD* = 4.79). Elite athletes were targeted for our sample as based on prior literature (Gould et al., 2002; Rees et al., 2016), we supposed that they will have high scores on perfectionism. Among the participants, there were one two-time Olympic gold medalist and several World and European Champions.

This research was approved by the Institutional Review Board of the local university. Participants first provided informed consent (and passive parental consent when necessary). They were informed that participation was voluntary and anonymous, without compensation. They then received the demographic and sports-related questions, which were followed by the Frost Multidimensional Perfectionism Scale (Frost et al., 1990), then the Sport Attributional Style Scale (SASS; Hanrahan et al., 1989), and finally the Short Self-Handicapping Scale (SHS; Rhodewalt, 1990).

### Measures

The Frost Multidimensional Perfectionism Scale (FMPS, Terry-Short et al., 1995) was used to measure perfectionistic concerns and striving. The FMPS is a 35-item self-report questionnaire originally designed to measure the multidimensional construct of perfectionism through six subscales (Personal Standards, Concern over Mistakes, Doubts about Actions, Parental Criticism, Parental Expectations, and Organization). It utilizes a five-point Likert-type scale ranging from “strongly disagree” (1) to “strongly agree” (5). Participants were asked to consider situations in sport indicating how strongly they agree with each statement (e.g., “Other people seem to accept lower standards from themselves than I do”). Following past research (Frost et al., 1993; Moser et al., 2012), we defined perfectionistic concerns to be composed of four subdimensions: Concern over Mistakes, Doubts about Actions, Parental Expectations, and Parental Criticism. Perfectionistic striving comprises two subdimensions: Personal Standards and Organization. In the present study, we found Cronbach’s coefficient alphas (*α*) of 0.90 for perfectionistic concerns and 0.82 for adaptive perfectionism.

The short 10-item SASS (Hanrahan et al., 1989; Hanrahan and Grove, 1990) was used to measure sport-related attributions. This scale presented 10 hypothetical situations (five positive and five negative). The positive and negative situations corresponded to each other and were matched for content (“The coach compliments/criticizes your performance”). Participants were asked to vividly imagine themselves in each situation and state what they believe would have been the single most likely cause of the given event. Then, participants were asked to rate their answer on a seven-point scale with five attributional dimensions: internality (internal vs. external), stability (stable vs. unstable), globality (global vs. specific), controllability (controllable vs. uncontrollable), and intentionality (intentional vs. unintentional).^1^ Based on previous research (e.g., Seligman et al., 1990; Martin-Krumm et al., 2003; Gordon, 2008), we totaled these scores separately for the negative and positive outcome situations providing separate composite scores for explanations of negative events (*α* = 0.72) and positive events (*α* = 0.71). Higher scores indicated internal, stable, global, controllable, and intentional attributions.

Self-reported self-handicapping tendencies, such as lack of effort, procrastination, and illness were assessed with the 14-item Self-Handicapping Scale (e.g., “I would do a lot better if I tried harder”; Rhodewalt, 1990). All items were assessed on a six-point scale ranging from “strongly disagree” (1) to “strongly agree” (6). The SHS score (*α* = 0.74) was formed by averaging the items; higher scores represented greater self-reported self-handicapping. Participants were asked to consider sport situations in connection with each item, indicating how strongly they agree with each statement.

### Analytic Strategy

In this study, SPSS 22.0 was used to analyze the data. We first examined correlations between the main variables and then conducted hierarchical regression analysis. In Step 1, we entered the scores for perfectionistic striving and concerns into SPSS. In Step 2, sport-related attributions (i.e., separate composite scores for explanations of negative events and of positive events) were entered to identify unique variance beyond the two forms of perfectionism. Finally, to test the hypothesis that attributions mediate the relationship between perfectionism and self-handicapping, we used the bootstrapping method recommended by Hayes (2017) to obtain bias-corrected 95% bootstrap CI for the indirect effects (Model 4).

## Results

### Zero-Order Correlations

Table 1 provides the descriptive statistics and correlations for each research variable. Self-handicapping showed a significant, positive correlation with perfectionistic concerns. On the other hand, the zero-order correlation was non-significant between self-handicapping and perfectionistic striving. In addition, self-handicapping displays a significant, positive correlation with the composite score for explanations of negative events, thus indicating that self-handicapping was associated with internal, stable, global, controllable, and intentional attributions for the negative events. Furthermore, perfectionistic concerns show a significant, positive correlation with both composite scores (i.e., explanations of negative and positive events). Participants characterized by high level of perfectionistic concerns tend to make internal, stable, global, controllable, and intentional attributions regardless of the valence of the event outcome (i.e., positive or negative situation). There was a nonsignificant association between the two types of perfectionism. Additionally, there was a significant correlation between the explanations of negative and positive events.

**Table 1:** *Means, SDs*, and zero-order correlations (*N* = 111).

Measure	1. Self-handicapping scale	2. Perfectionistic concerns	3. Perfectionistic striving	4. Explanations of negative events	5. Explanations of positive events
1. Self-handicapping scale	−				
2. Perfectionistic concerns	0.34^**^	−			
3. Perfectionistic striving	−0.04	0.12	−		
4. Explanations of negative events	0.37^**^	0.35^**^	−0.06	−	
5. Explanations of positive events	0.12	0.39^**^	0.12	0.62^**^	−
*Mean*	41.62	2.44	3.86	107.83	108.26
*SD*	9.94	0.71	0.60	14.69	13.46

Explanations of negative events = composite score for explanations of negative events, Explanations of positive events = composite score for explanations of positive events.

** *p* < 0.01, two-tailed tests.

### Regression Analysis

Multicollinearity (indicated by VIF > 10 and/or tolerance < 0.10) was not detected in the regression analysis (Myers, 1990). Perfectionistic concerns were a significant, positive predictor of self-handicapping, whereas perfectionistic striving was a significant, negative predictor of self-handicapping in both Step 1 and Step 2, which supports our hypotheses. After the sport-related attributions were added to the model in Step 2, the standardized betas of perfectionistic concerns and striving weakened but remained significant. In Step 2, the composite score of the explanations for negative events was a significant predictor of self-handicapping: those participants who adopted internal, stable, global, controllable, and intentional attributions for the negative events were more likely to show self-handicapping tendencies. Similarly, the explanations for positive events was a significant negative predictor of self-handicapping: those participants who adopted internal, stable, global, controllable, and intentional attributions for the positive events were less likely to show self-handicapping tendencies (see Table 2).

**Table 2:** Hierarchical regression analysis predicting self-handicapping.

	DV: Self-handicapping							
	Step 1			Step 2				
	*β*	*SE*	*β* 95% CI	*p*	*β*	*SE*	*β* 95% CI	*p*
Perfectionistic concerns	0.52	1.40	[4.94, 10.47]	<0.001	0.46	1.48	[3.95, 9.83]	<0.001
Perfectionistic striving	−0.27	1.75	[−8.56, −1.61]	0.01	−0.20	1.74	[−7.21, −0.29]	0.03
Explanations of negative events					0.34	0.07	[0.08, 0.38]	0.002
Explanations of positive events					−0.24	0.08	[−0.34, −0.02]	0.03
	Model summary *R*^2^ = 0.231, *F*(2, 106) = 15.62, *p* < 0.001			Model summary *R*^2^ = 0.300, *F*(4, 106) = 10.91, *p* < 0.001				

Explanations of negative events = composite score for explanations of negative events, Explanations of positive events = composite score for explanations of positive events. *β*, standardized coefficients.

### Multiple Mediation Analysis

Bootstrapping (Hayes, 2017) was used in order to test the hypotheses that sport-related attributions mediate the relationship between the two forms of perfectionism and self-handicapping. To assess the independent indirect effects of these relationships, we used bootstrapping to obtain the bias-corrected 95% CI for the total indirect effects of both forms of perfectionism and the specific indirect effects of each mediator.

**Figure 1:**
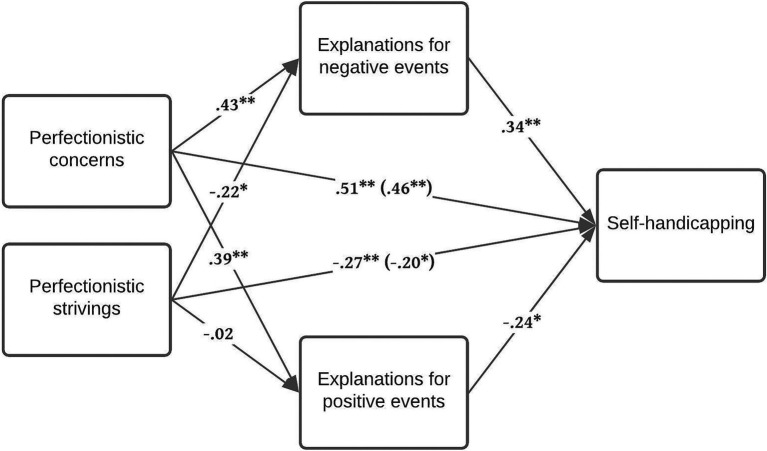
Explanations for positive and negative events as mediators of the effect of the adaptive (striving) and the maladaptive (concerns) aspects of perfectionism on self-handicapping tendencies. Path values represent standardized coefficients. ^*^p < 0.05, ^**^p < 0.01.

The total indirect effect of perfectionistic concerns *via* sport-related attributions on self-handicapping was non-significant, *b* = 0.06, *SE* = 0.05, 95% CI [−0.04, 0.16]. However, the scores for explanations of positive events and scores for explanations of negative events cancel each other out, leading to this non-significant total indirect effect (see Demming et al., 2017). In fact, both the composite score for explanations of negative events, *b* = 0.16, *SE* = 0.07, 95% CI [0.04, 0.30] and the composite score for explanations of positive events, *b* = −0.10, *SE* = 0.06, 95% CI [−0.23, −0.01] were significant mediators between perfectionistic concerns and self-handicapping. The total direct (unmediated) effect of perfectionistic concerns on self-handicapping, *b* = 0.50, *SE* = 0.11, 95% CI [0.28, 0.71], as well as the total effect, *b* = 0.55, *SE* = 0.10, 95% CI [0.35, 0.75] were significant (Sobel tests: *z* = 2.24, *p* = 0.03 for the negative events; *z* = −0.42, *p* = 0.67 for the positive events). The model is depicted in Figure 1.

The total indirect effect of adaptive perfectionism *via* sport-related attributions on self-handicapping was negative and significant, *b* = −0.08, *SE* = 0.04, 95% CI [−0.17, −0.01]. Examination of the bias-corrected 95% CI from 5,000 bootstrap samples revealed that the composite score for explanations of positive events was not a significant mediator, *b* = 0.01, *SE* = 0.03, 95% CI [−0.05, 0.07]. However, the composite score for explanations of negative events was, *b* = −0.09, *SE* = 0.05, 95% CI [−0.19, −0.01], which supports our hypothesis (see Figure 1). Both the total direct (unmediated) effect of adaptive perfectionism on self-handicapping, *b* = −0.23, *SE* = 0.11, 95% CI [−0.43, −0.02], and the total effect, *b* = −0.31, *SE* = 0.11, 95% CI [−0.52, −0.10] were significant (Sobel tests: *z* = −0.63, *p* = 0.53 for the negative events; *z* = 0.92, *p* = 0.36 for the positive events).

## Discussion

Athletes differ in their extents of self-handicapping across the dimensions of perfectionism, and their beliefs regarding negative sports events play a significant role in determining this. The purpose of this study was to examine a model linking self-handicapping with perfectionism through the mediation of attributional styles among elite athletes. The results showed significant relationships between the variables in the directions hypothesized. As it was expected, a positive relationship was found between perfectionistic concerns and self-handicapping, whereas there was a negative relationship between perfectionistic striving and self-handicapping. Preoccupation with avoiding mistakes (in contrast to approaching success) is a common base of both perfectionistic concerns and self-handicapping. By contrast, adaptive perfectionism is characterized by a desire to strive for excellence deriving from an approach motivational basis (Elliot and Church, 2003; Gucciardi et al., 2012; Burnam et al., 2014). Individuals that display characteristics of perfectionistic concerns may resort to self-handicapping due to the need to protect themselves against the threat of failure as a result of setting unattainable goals.

Our results showed that perfectionistic concerns were positively related to interpreting positive events as internal, stable global, controllable, and intentional. Similarly to previous studies (e.g., Harper et al., 2020), we found that perfectionistic concerns were positively associated with self-depreciating attributional style (internal, stable, global, controllable, and intentional) for negative events. This result can be a consequence of elevated self-blame that people with high level of maladaptive perfectionism experience (Stoeber and Janssen, 2011). Therefore—with using a composite score of attributions—we might see a strong sensitivity to both positive and negative feedback that can explain the self-depreciating attributions after negative feedback as well as the self-enhancing reactions to positive feedback. These results indicate that elite athletes with high perfectionistic concern could be particularly reliant on feedback and take them especially seriously irrespective of their direction. Perfectionistic striving and self-depreciating attributional style regarding negative events show an inverse relationship. This means that participants who scored higher on the adaptive aspects of perfectionism made less internal, stable, global, controllable, and intentional attributions for the negative events. This result supports previous findings showing that people with high perfectionistic striving tend to attribute failures in a self-favoring way (e.g., Levine et al., 2017). This kind of attributional style may lead to adaptive functioning in performance settings that is a characteristic of healthy perfectionistic individuals (Gould et al., 2002). Our results also support the theory of optimal illusions (Baumeister, 1989; see also Brown, 2014) showing that distorting the reality toward the positive end may lead to elevated well-being and more adaptive functioning.

According to our results, the relationship between both perfectionistic aspects and self-handicapping is partially mediated by the attributional style in connection with negative events. More specifically, those displaying internal, stable, global, controllable, and intentional attributions when presented with negative events were more likely to show self-handicapping tendencies; this kind of attributional style is particularly related to perfectionistic concerns. The same attributional style regarding positive events has been associated with perfectionistic concerns, but this was not found to be related to self-handicapping. This is partly in line with previous research (Rhodewalt, 1990; Anshel and Mansouri, 2005); however, it also broadens our theoretical and empirical understanding of the mediating role of attributions between perfectionism and self-handicapping. Our results showed that explanations for negative events are crucial in connection with protecting oneself *via* self-handicapping among top athletes. Experiencing self-blame and rumination on failures deriving from excessively high standards may lead to punctual maladaptive attributions and chronic maladaptive attributional style in the long run regarding performance-events (Flett et al., 2002; Stoeber and Janssen, 2011; Harper et al., 2020). Since this kind of thinking poses a threat to one’s self-esteem (e.g., fixed implicit theory of ability; Chen et al., 2008), it could result in more intense self-protection such as self-handicapping, leading to a vicious circle.

However, these findings may have some limitations. First, the constructs investigated in this study are self-reported in nature, thus it is not clear what role self-deception plays in these processes (Török et al., 2018). In the future, more objective methods (such as behavioral measures) could be employed. Second, the measure used for attributional styles is sport-specific, while the measures we implemented to assess perfectionism and self-handicapping were not specific to sports situations—although we asked participants to take into account sport-relevant situations while answering the questions. Future research should consider this factor since both constructs could differ across domains (Stoeber and Becker, 2008; Schwinger, 2013; Sellars et al., 2016). Third, although the sample size was special in terms of the level of the performers, it was numerously small. Therefore, further larger studies are required to confirm these results. It should also be noted that the results obtained in this study are based on correlational relationships, so further investigations are needed to clarify causal interpretations.

Nonetheless, the present findings have important implications in order to understand the processes involved with perfectionism and self-handicapping in sports. This study provides further support for the multidimensional nature of perfectionism by identifying adaptive and maladaptive correlates of the construct (Frost et al., 1993; Rice and Ashby, 2007; Hill et al., 2020). As a coach, it is crucial to maintain a comprehensive stance on perfectionism that considers its positive and negative aspects for individual athletes. Our results provide further support to prior studies demonstrating that attributional retraining techniques could be beneficial in achievement settings (Perry et al., 2010) such as sport (Rascle et al., 2008; Parkes and Mallett, 2011). Furthermore, the obtained results are based on a sample of top performing elite athletes, which is very sparse in the literature within this field (but see Gould et al., 2002; Kuczka and Treasure, 2005). The majority of the sample were top-level athletes including Olympic medalists and a world record holder. Interestingly, self-handicapping was clearly present even in this sample. It appears that the level of self-handicapping in our sample was relatively similar to other samples (e.g., Ryska, 2002; Prapavessis et al., 2003; Allen, 2018, Unpublished manuscript^2^). Although, the most successful athletes from our sample, such as Olympic and World or European Championships medalists (*N* = 30) showed significantly lower self-handicapping (*M* = 37.69, *SD* = 9.29) compared to the rest of the sample (*M* = 43.18, *SD* = 10.10). Future research should consider perfectionism according to the 2 × 2 model of Gaudreau and Thompson (2010) and extend the current model with non-perfectionism (low in both adaptive and maladaptive aspects) and mixed perfectionism (high in both adaptive and maladaptive aspects) in order to gain a better understanding of these processes.

## Data Availability Statement

The raw data supporting the conclusions of this article will be made available by the authors, without undue reservation.

## Ethics Statement

The studies involving human participants were reviewed and approved by Eotvos Lorand University, Faculty of Education and Psychology Institutional Review Board, 2018/413. Written informed consent to participate in this study was provided by the participants’ legal guardian/next of kin.

## Author Contributions

LT and ZS contributed to the study design, literature review, and data gathering. LT, ZS, and GO contributed to the manuscript writing, data analysis, and interpretation. All authors contributed to the article and approved the submitted version.

## Funding

GO was supported by the Young Researcher STARS grant from Conseil Régional Hauts de France and by the Strategic Dialogue and Management Scholarship (Phase 2).

## Conflict of Interest

The authors declare that the research was conducted in the absence of any commercial or financial relationships that could be construed as a potential conflict of interest.

## Publisher’s Note

All claims expressed in this article are solely those of the authors and do not necessarily represent those of their affiliated organizations, or those of the publisher, the editors and the reviewers. Any product that may be evaluated in this article, or claim that may be made by its manufacturer, is not guaranteed or endorsed by the publisher.

## Footnotes

^1^The participants were also requested to indicate on seven-point scales the importance and the vividness of the event. These are not attributional dimensions; therefore, we did not include these measures in the present analytic model.

^2^Allen, J. (2018). Burnout, Motivation, and Self-Handicapping in Collegiate Club Athletes. Unpublished manuscript. Bowling Green State University, Bowling Green, USA.
